# Sensing Region Characteristics of Smart Piezoelectric Interface for Damage Monitoring in Plate-Like Structures

**DOI:** 10.3390/s19061377

**Published:** 2019-03-19

**Authors:** Thanh-Canh Huynh, So-Young Lee, Ngoc-Loi Dang, Jeong-Tae Kim

**Affiliations:** 1 BK21Plus Team, Department of Ocean Engineering, Pukyong National University, 599-1 Daeyeon 3-dong, Nam-gu, Busan 608-737, Korea; ce.huynh@gmail.com (T.-C.H.); loi.ngocdang@gmail.com (N.-L.D.); 2 Smart Infrastructure Technology Institute, Pukyong National University, 599-1 Daeyeon 3-dong, Nam-gu, Busan 608-737, Korea; soyounglee.amy@gmail.com

**Keywords:** impedance-based method, electromechanical impedance, PZT interface, damage sensitivity, sensing region, plate structure

## Abstract

For impedance-based damage detection practices, the sensing range of piezoelectric devices is an important parameter that should be determined before real implementations. This study presents numerical and experimental analyses for characterizing the sensing region of a smart PZT (lead–zirconate–titanate) interface for damage monitoring in plate-like structures. First, a finite element (FE) model of the PZT interface mounted on a plate structure is established. The impedance responses of the PZT interface are numerically simulated under different damage locations inflicted in the plate domain. The impedance features are extracted from the impedance signatures to analyze the sensing distance and the damage detectability of the PZT interface. Next, the splice plate of a bolted connection is selected as a practical plate-like structure for the experimental examination of the PZT interface’s sensing region on a limited plate domain. The damage sensitivity behavior of the PZT interface is analyzed with respect to the damage location on the splice plate. An FE analysis of the corresponding PZT interface-splice plate system is also conducted to support the experimental results.

## 1. Introduction

The impedance-based damage diagnosis in local critical members of civil infrastructures has been received significant attention by many researchers over the past decades [1,2,3,4,5,6,7,8,9,10,11,12] To detect structural damage, the host structure is instrumented with the PZT (lead–zirconate–titanate) patch, which is electrically excited at high frequencies. The electromechanical (EM) impedance, which is resulted from the coupled vibrations between the PZT patch and the host structure, is then extracted. The early theoretical model of coupled EM impedance measurements was presented by Liang et al. [1]. Based on the Liang’s model, it is demonstrated that any structural damage occurred in the host structure will lead to the variation of the EM impedance responses that can be quantified using statistical index metrics.

An issue of the conventional impedance-based technique is on setting effective frequency bands which should be sensitive to the changes in structural parameters of the host structure. In practice, the effective frequency bands for given target structures are traditionally identified by the trial-and-error method [13]. The second issue is the need of high-performance and expensive impedance analyzers to precisely record the impedance in extreme high-frequency bands which often contains the information of incipient damages. Park et al. [13] reported that the frequency bands higher than 200 kHz are found to be favorable for a local sensing area while the ones lower than 70 kHz covers a larger sensing area. Soh et al. [14] performed the impedance-based health monitoring in a reinforced concrete bridge and showed that the effective frequency band was within 120–625 kHz. By conducting impedance monitoring in the bearing plate of a prestressed tendon-anchorage, Kim et al. [15] observed that the effective frequency band was even over 800 kHz.

The PZT interface technique was developed as an alternative way to overcome these above-mentioned issues. Huynh and Kim [16] proposed a portable PZT interface device, which is an aluminum beam-like structure embedded with a PZT sensor. The interface structure has a middle flexible beam section and the two outside contact surfaces, which allows the interface to be easily mounted on and detached from the target structure, thus enabling convenient reconfiguration of the device in necessary cases [17]. The PZT interface technique can reduce the need of high-performance impedance analyzers, and allow predetermining the effective frequency bands. The experimental feasibility of the PZT interface-based impedance monitoring technique were evaluated under constant temperatures. To deal with the impedance monitoring under the effects of environmental factors, researchers have developed principal component analysis-based, artificial radial basis function network-based, and effective frequency shift-based algorithms [18,19].

For impedance-based damage detection practices, the sensing range of piezoelectric sensors is an important parameter that should be determined before real implementations [13]. The sensing range of impedance signatures is of significant interest for the proper sensor placement on the target structure [20,21]. However, this important research is often ignored in both practice and literature in piezoelectric-based structural health monitoring technology. So far, there has been very little research on determining the piezoelectric sensor’s sensing region for the impedance-based method. At high-frequency ranges, the sensing region of the piezoelectric sensor is localized to a small region, which is close to the sensor [22]. Esteban [23] conducted theoretical modeling efforts based on the wave propagation approach to identify the PZT’s sensing region. Based on the knowledge acquired via various case studies, Park et al. [24] reported that (depending on the material and density of the structure) the sensing area of a single PZT can vary anywhere from 0.4 m (sensing radius) on composite reinforced concrete structures to 2 m on simple metal beams.

This study presents numerical and experimental analyses for characterizing the sensing region of a smart PZT (lead–zirconate–titanate) interface with a focus on damage monitoring in plate-like structures. The study aims to give a guidance for installing the PZT interface and choosing frequency ranges in fault detection processes. First, a finite element (FE) model of the PZT interface mounted on a plate structure is established. The impedance responses of the PZT interface are numerically simulated under different damage locations inflicted in the plate domain. The impedance features are extracted from the impedance signatures to analyze the sensing distance and the damage detectability of the PZT interface. Next, the splice plate of a bolted connection is selected as a practical structure for the experimental evaluation of the PZT interface’s sensing region on a limited plate domain. The damage sensitivity behavior of the PZT interface is analyzed with respect to the damage location on the splice plate. An FE analysis of the corresponding PZT interface-splice plate system is also conducted to support the experimental results.

## 2. Piezoelectric Interface-Based Impedance Monitoring Technique

### 2.1. Impedance Monitoring via Piezoelectric Interface

**Figure 1:**
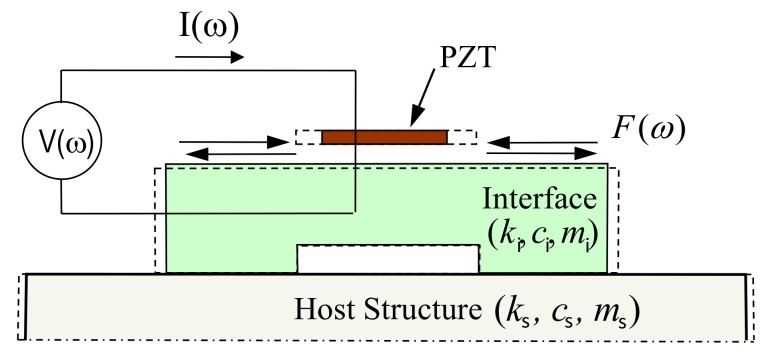
PZT (lead–zirconate–titanate) interface-host structure interaction under voltage excitation.

The PZT interface technique was proposed to alternatively monitor impedance signatures by predetermining sensitive frequency bands of the target structure [16]. Figure 1 shows an interaction between the PZT interface (represented by *k_i_, c_i_,* and *m_i_*) and a host structure (represented by *k_s_, c_s_,* and *m_s_*). The interface is designed with two outside bonded sections for the attachment. The middle part of the interface is designed with a flexible section to provide free vibrations for the PZT sensor.

To acquire the impedance responses, a harmonic voltage *V*(*ω*) is applied to the PZT sensor and the output electric current *I*(*ω*) is then measured by an impedance analyzer. The EM impedance of the whole system, *Z*(*ω*), is a combined function of the structural mechanical (SM) impedance of the piezoelectric patch, *Z_a_*(*ω*), and that of the coupled interface-host structure $\bar{Z}\left(\omega \right)$, as described below:

(1) $$Z\left(\omega \right)=\frac{V}{I}={\left\{i\omega \frac{w_{a}l_{a}}{t_{a}}\left[{\hat{\varepsilon }}_{33}^{T}-\frac{1}{Z_{a}\left(\omega \right)/\bar{Z}\left(\omega \right)+1}d_{31}^{2}{\hat{Y}}_{11}^{E}\right]\right\}}^{-1}$$

where ${\hat{Y}}_{xx}^{E}=\left(1+i\eta \right)Y_{xx}^{E}$ is the complex Young’s modulus of the PZT patch at zero electric field; ${\hat{\varepsilon }}_{xx}^{T}=\left(1-i\delta \right)\varepsilon_{xx}^{T}$ is the complex dielectric constant at zero stress; d_3x_ is the piezoelectric coupling constant in *x*-direction at zero stress; and *w_a_*, *l_a_*, and *t_a_* are the width, length, and thickness of the PZT patch, respectively. The parameters *η* and *δ* are structural damping loss factor and dielectric loss factor of piezoelectric material, respectively. The SM impedance of the coupled interface-host structure system $\bar{Z}\left(\omega \right)$ can be computed from the following equation [25]:

(2) $$\bar{Z}\left(\omega \right)=\frac{\left(-\omega^{2}m_{s}+i\omega c_{i}+k_{i}\right)(-\omega^{2}m_{s}+i\omega \left(c_{i}+c_{s}\right)+\left(k_{i}+k_{s}\right)-{\left(i\omega c_{i}+k_{i}\right)}^{2}}{i\omega \left(-\omega^{2}m_{s}+i\omega \left(c_{i}+c_{s}\right)+\left(k_{i}+k_{s}\right)\right)}$$

Equation (2) shows that the SM impedance of the coupled interface-host structure system is characterized by the masses, damping, and stiffness of both the interface (*k_i_, c_i_, m_i_*) and the host structure (*k_s_, c_s_, m_s_*). Thus, the change in these parameters caused by environmental factors and structural damage can be represented by the change in the EM impedance obtained from the PZT sensor. By quantifying the variations of EM impedance signals, the structural damage occurred in the host structure can be detected.

### 2.2. Damage Classification Using Impedance Signatures

For damage quantification, two well-known statistical damage indices: root-mean-square-deviation (RMSD) and correlation coefficient deviation (CCD) are usually used. The RMSD index is defined, as follows [2]:

(3) $$\mathrm{RMSD}\left(Z,Z^{*}\right)=\sqrt{\frac{\sum_{i=1}^{N}{\left[Z^{*}\left(\omega_{i}\right)-Z\left(\omega_{i}\right)\right]}^{2}}{\sum_{i=1}^{N}{\left[Z\left(\omega_{i}\right)\right]}^{2}}}$$

where $Z\left(\omega_{i}\right)$ and $Z^{*}\left(\omega_{i}\right)$ are the EM impedances measured before and after a damage scenario for the ith frequency, respectively; and N denotes the number of frequency points in the sweep.

The CCD index can also be used to quantify the change in the impedance signatures [3]. The CCD index is calculated, as follows:

(4) CCD=1−1σzσz*EReZi−ReZ¯ReZi*−ReZ¯*

where E[.] is the expectation operation; ReZi signifies the real parts of the EM impedances of the ith frequency before and after damage; ReZ¯ signifies the mean values of impedance signatures (real part) before and after damage; and $\sigma_{z}$ signifies the standard deviation values of impedance signatures before and after damage. Note that the asterisk (*) denotes the damaged state.

Basically, the damage indices (i.e., RMSD and CCD) are equal to 0 if no damage and larger than 0 if damage occurs. However, due to experimental and computational errors, the damage indices may be larger than 0 although damage is not occurred. To minimize the effect caused by the uncertain conditions, the control chart analysis is used for decision-making. The upper control limit (UCL) of the damage index is adopted for alarming damage occurrence, as follows:

(5) UCL=μ+3σ

where *μ* and *σ* are mean and standard deviation of the damage index data at undamaged condition, respectively. In Equation (5), the UCL is determined by three standard deviations of the mean, which is corresponding to 99.7% confidence level.

## 3. Sensing Region Characteristics of PZT Interface on a Numerical Plate Domain

### 3.1. FE Modeling

The sensing region of the PZT interface was characterized for a numerical plate domain to determine the sensitive zones in the damage detection. An FE model of the PZT interface mounted on the plate domain was established using COMSOL Multiphysics (COMSOL Inc., Burlington, MA, USA). The FE modeling of piezoelectric effects of the PZT interface requires multiple coupled physics (i.e., electrical and mechanical), which act simultaneously during the excitation of the sensor. Due to its electrical-mechanical simulation capabilities which have been widely accepted by the academic society, COMSOL Multiphysics was selected to simulate the piezoelectric effects [7,26,27,28].

**Figure 2:**
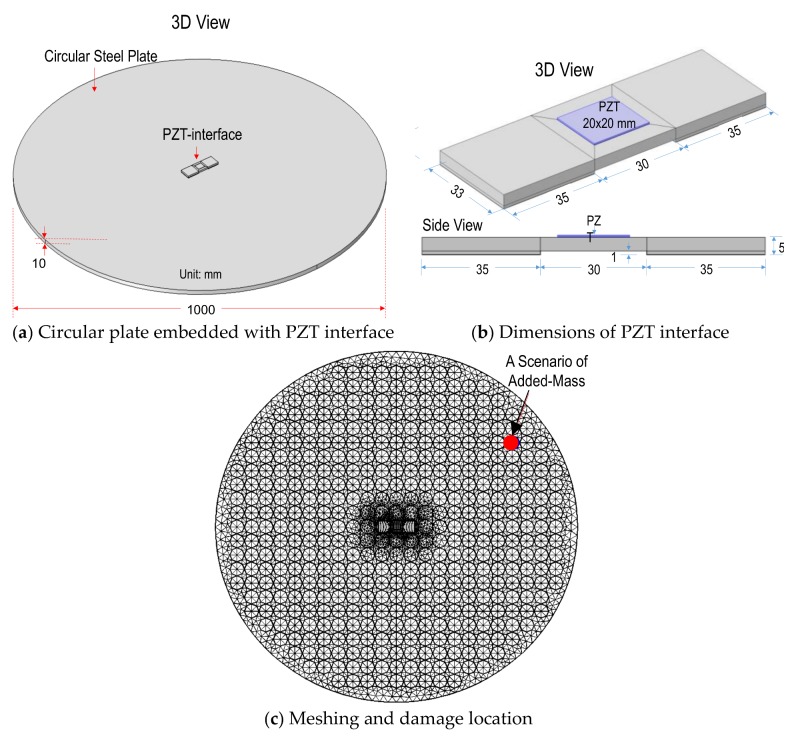
Finite element (FE) modeling for analyzing sensing region of PZT interface on a plate domain.

As shown in Figure 2a, the circular plate has a 1 m diameter and a 0.01 m thickness. The plate has orders of a geometric size larger than the PZT interface. As detailed in Figure 2b, the PZT interface as the following geometric parameters: 33 × 35 × 5 mm^3^ for two outside bonded sections and 33 × 30 × 4 mm^3^ for flexible section. The flexible section was embedded with a PZT-5A sensor with the sizes of 20 × 20 × 0.51 mm^3^, see Figure 2b. The bonding layers of the PZT sensor and the interface have the thickness of 0.1 mm. The properties of the bonding layers were selected as listed in Table 1 [29,30]. The interface body, the plate domain, and the PZT were discretized by 3D block elements. Specifically, hexahedron elements were used for meshing the PZT sensor and the interface body while tetrahedron elements were used for the circular plate. To activate the piezoelectric effects, the PZT sensor was simulated using the piezoelectric elements which can deal with both mechanical and electrical fields. As shown in Figure 2c, a complete mesh of the FE model consists of 8618 solid elements. Free boundary conditions were applied for the FE model. The material properties of the steel plate domain, the aluminum interface and the bonding layers, which specified in the FE model, are listed in Table 1 [31]. The piezoelectric properties of the PZT patch (PZT-5A type) can be found in Huynh et al. [31] and efunda Inc. [32].

Due to the simplification and computational cost-effectiveness in simulating damage scenarios, a damage simulation technique using an added-mass instead of real damage was employed to study the sensing region of the PZT interface on the plate domain. The use of added mass to physically model the stiffness-loss has been widely accepted by many researchers [15,33,34]. It is noted that the added mass simulation could represent the corrosion-type damage in reality, but may not replace the crack-type damage. Accordingly, 4 cm grid was designed for the assignment of the added-mass of 0.174 kg on the plate (see Figure 2c). Each intersection of the grid was corresponding to a specific stiffness-loss scenario simulated in the testing plate. Due to the symmetry of the target structure, only a quarter of the plate was assigned by the added-mass. Totally, 121 scenarios of the stiffness-loss were simulated over a quarter of the testing plate. To acquire the impedance impedance responses, harmonic excitation of 1V was applied to the PZT sensor and the frequency was swept in the frequency range of 10–55 kHz (901 interval points).

### 3.2. Numerical Impedance Responses

**Figure 3:**
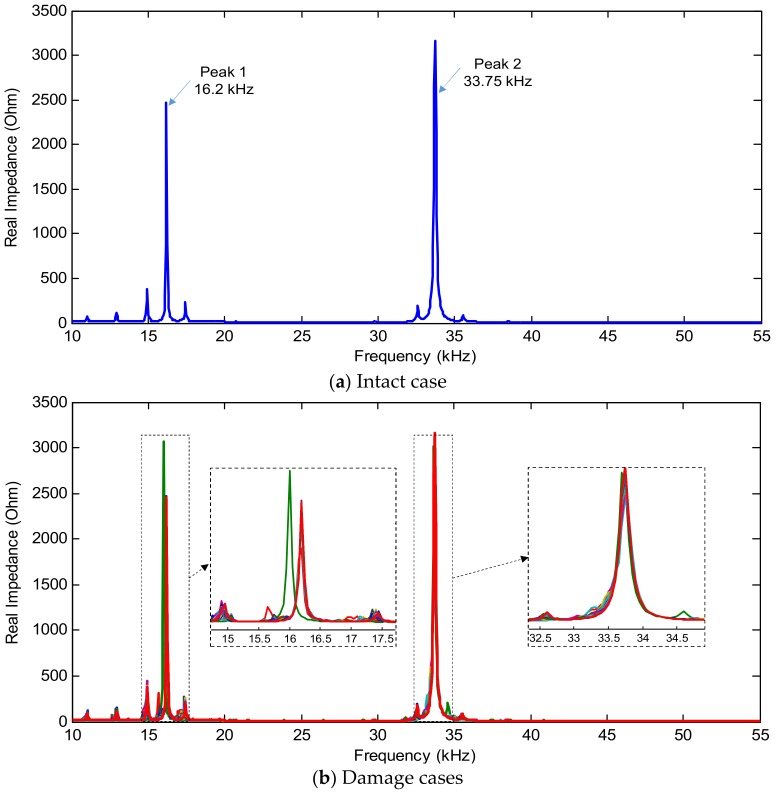
Numerical impedance signatures of PZT interface for intact and damage cases of plate domain.

The impedance responses of the PZT interface are numerically simulated under different damage locations inflicted in the plate domain. Figure 3 shows the numerical impedance responses in the frequency range of 10–55 kHz for the coupled PZT interface-plate system under the intact and damage cases. Two major resonant ranges 10–20 kHz and 30–40 kHz are observed within the examined frequency range of 10–55 kHz. Under the intact case, Peak 1 and Peak 2 were identified at 16.2 kHz and 33.75 kHz, respectively (see Figure 3a). When the mass was added to the circular plate, it is observed that the peak frequencies varied sensitively (see Figure 3b). The resonant range 10–20 kHz changed more sensitively than 30–40 kHz under the stiffness-loss cases (see Figure 3b).

**Figure 4:**
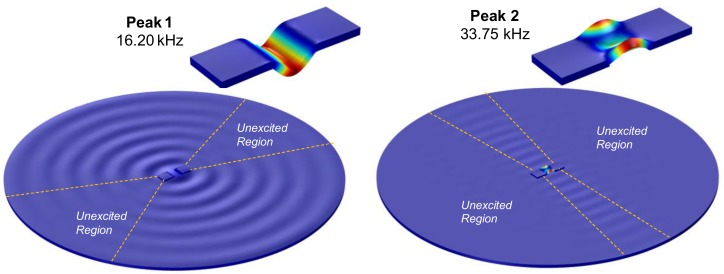
Coupled vibration modes of the PZT interface-plate domain system.

To give an overview on the wave propagation through the plate domain, the coupled vibration of the PZT interface-circular plate was simulated for the two peak frequencies (Peaks 1 and 2). As observed in Figure 4, the motion of Peak 1 matched to the longitudinal flexural mode while that of Peak 2 was consistent with the lateral flexural mode. These flexural motions generated high-frequency vibrational waves which propagated from the center toward the boundaries of the circular plate. There exists fan-shaped regions (i.e., unexcited regions) where the vibrational waves were very weak on the circular plate. Because of the weak coupling responses between the interface and the host structure, structural damage occurred in these unexcited regions will be hard to be detected. As compared to Peak 1, the area of the unexcited regions corresponding to Peak 2 was larger. The above analysis of the wave propagation can overall identify the zones on the plate domain sensitive to the structural damage.

### 3.3. Numerical Characterization of Sensing Region

#### 3.3.1. Sensitivity of Impedance Features with Respect to Damage Location

**Figure 5:**
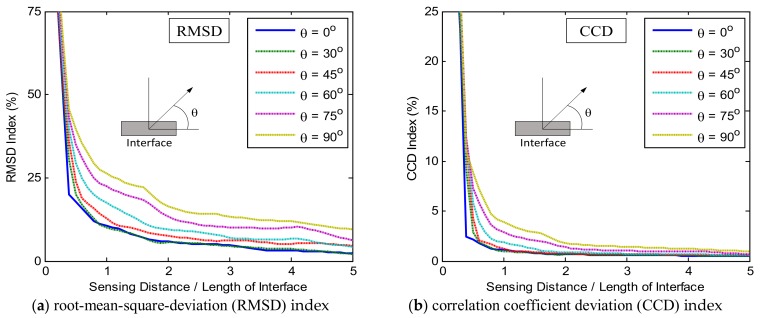
Relationships between damage-sensitive impedance features and sensing distance: Peak 1 in 10–20 kHz.

The impedance features (RMSD and CCD) were extracted from the impedance signatures to analyze the damage sensitivity of the PZT interface with respect to the damage detection on the plate domain. Figure 5 shows the relationships between the damage-sensitive impedance features (i.e., RMSD and CCD) and the sensing distance for the frequency range of 10–20 kHz (Peak 1). In the figure, *θ* is the angle between the radius vector and the interface’s longitudinal axis, and the sensing distance was normalized to the interface’s length. As shown in Figure 5a,b, the magnitudes of the RMSD and CCD indices increased when the angle *θ* changed from 0° to 90°. The changes in these damage indices was found to be ignorable for the angles ranging from 0° to 30°. The most favorable angle for the damage detection was found at 90° where the magnitudes of the RMSD and CCD indices reached the maximum. As the sensing distance was increased, the magnitudes of the damage-sensitive indices went down. The CCD magnitudes were found to be ignorable after only a distance of two times of the interface’s length (see Figure 5b). Meanwhile, the RMSD magnitudes corresponding to the angle around 90° remained relatively significant after a distance of five times of the interface’s length (see Figure 5a).

**Figure 6:**
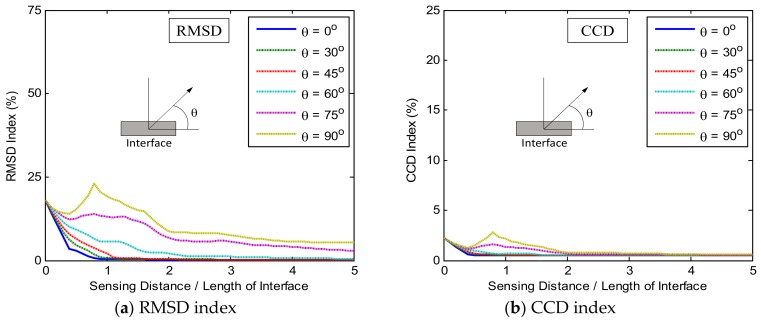
Relationships between damage-sensitive impedance features and sensing distance: Peak 2 in 30–40 kHz.

Figure 6 shows the relationships between the damage-sensitive impedance features (i.e., RMSD and CCD) and the sensing distance for the frequency range of 30–40 kHz (Peak 2). Similar to the previous observations, the magnitudes of the RMSD and CCD indices increased when the angle *θ* changed from 0° to 90°. The changes in these damage indices was found to be ignorable for the angle *θ* in 0°−30°, and the most favorable angle for the damage detection was found at 90°. As the sensing distance was increased, the magnitudes of the damage-sensitive indices went down. It is observed that after a distance of two times of the interface’s length, the magnitudes of the RMSD and CCD indices were ignorable (see Figure 6a,b).

#### 3.3.2. Detectable Zone of Piezoelectric Interface

**Figure 7:**
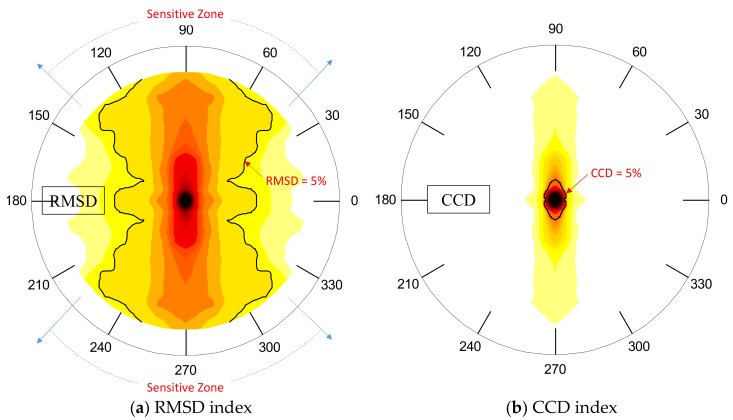
Detectable zone of PZT interface over circular plate domain: Peak 1 in 10–20 kHz.

To identify the detectable region of the PZT interface, the damage-sensitive impedance features (i.e., RMSD and CCD) were computed over the plate domain. Figure 7 shows the filled contours of the RMSD and CCD for the frequency range of 10–20 kHz (Peak 1). The contour lines of 5% were set as the threshold for the damage detection. The value of 5% was selected by considering the uncertain errors that may occur in real applications. The region containing the damage indices larger than 5% was classified as ‘detectable zone’. The detectable zones corresponding to the RMSD index were big fan-shaped regions (see Figure 7a) while those for the CCD index were only a small diamond-shaped zone (see Figure 7b).

**Figure 8:**
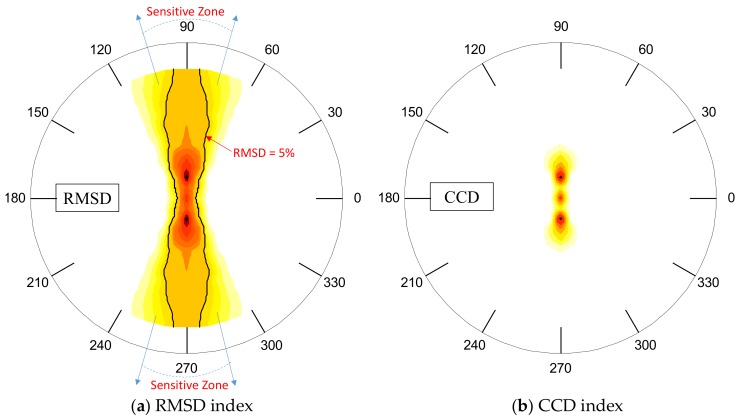
Detectable zone of PZT interface over circular plate domain: Peak 2 in 30–40 kHz.

Figure 8 shows the filled contours of the RMSD and CCD for the frequency range of 30–40 kHz (Peak 2). As observed in Figure 8a, the detectable zones corresponding to the RMSD index were narrow fan-shaped regions. Meanwhile, there were no detectable regions found in Figure 8b because the CCD magnitudes over the plate domain was less than the threshold of 5%. As compared with Peak 1 in 10–20 kHz, the detectable zones of Peak 2 in 30–40 kHz were much smaller. This means that the frequency range of Peak 1 should be utilized for damage detection.

Using the contours of the RMSD index, the favorable angle *θ* for damage detection on the circular plate domain can be approximately interpreted as follows: 70° ~110° and 250° ~ 290° for the range 10–55 kHz; 45° ~ 135° and 225° ~315° for the range 10–20 kHz; and 75° ~105° and 255° ~295° for the range 30–40 kHz.

#### 3.3.3. Damage Detectability with Respect to Plate’s Thickness

**Figure 9:**
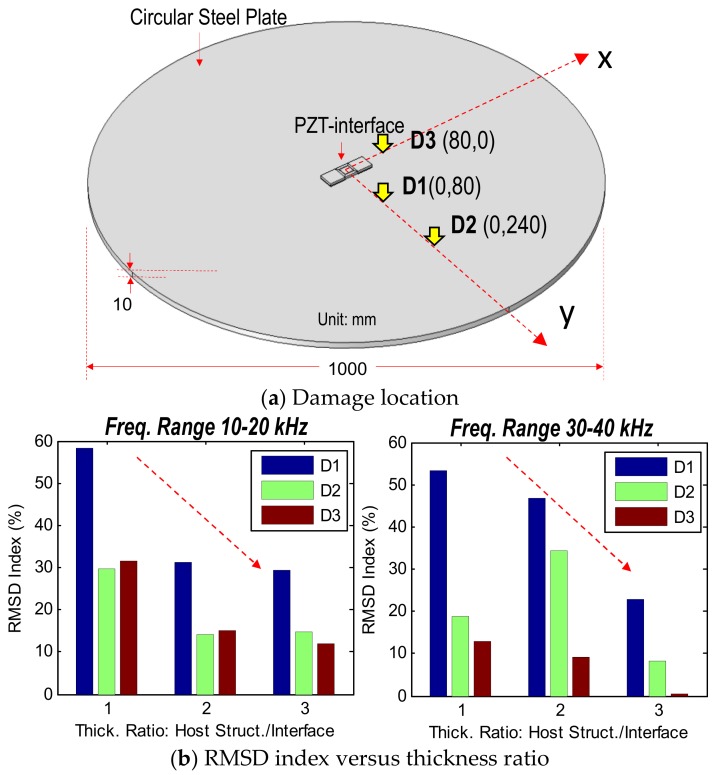
Damage detectability of PZT interface’s impedance signatures with respect to plate’s thickness.

The damage detectability of the PZT interface on the plate domain was analyzed with respect to their thickness ratio. The thickness of the interface was kept as constant as 5 mm while the thickness of the plate was increased from 5 to 10 and 15 mm. This means the thickness ratio between the host structure and the interface was ranged from 1 to 2 and 3. Figure 9a shows three damage locations (i.e., D1–D3) simulated in the plate domain. The RMSD index of the three frequency ranges (i.e., 10–55 kHz, 30–40 kHz, and 10–20 kHz) was computed and plotted according to the thickness ratio, as shown in Figure 9b. It is observed that the damage detectability of the interface was decreased as the thickness of the plate was increased.

## 4. Sensing Region Characteristics of PZT Interface on a Realistic Plate Structure

### 4.1. Test-Setup of Connection Splice Plate

**Figure 10:**
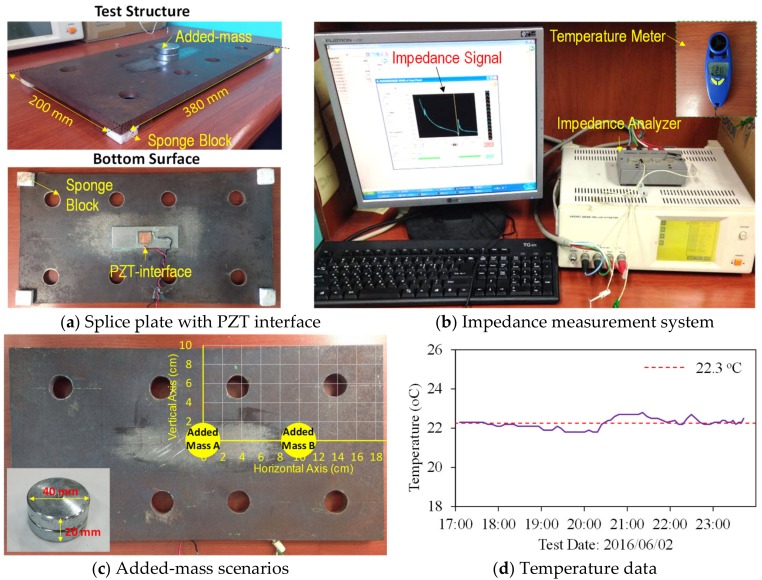
Test-setup for analyzing sensing region of PZT interface on a connection splice plate.

The splice plate of a bolted connection was selected as a practical plate-like structure for the experimental evaluation of the PZT interface’s sensing region on a limited plate domain. As shown in Figure 10a, the test structure is a rectangular-shaped plate of 0.2 m × 0.38 m with a thickness of 0.01 m. Four small sponge blocks with an ignorable modulus of elasticity were used to support the splice plate. A PZT interface was mounted at the bottom middle of the splice plate. The PZT interface has the same specifications with the one simulated in Section 3.1. The PZT patch was excited by a harmonic excitation of 1V-amplitude, and the impedance signal was measured in 10–55 kHz (901 points) using the impedance analyzer HIOKI 3532, as illustrated in Figure 10b. A handheld temperature meter (TFA Dostmann) was used to measure the room temperature during experimental tests, see Figure 10b.

As shown in Figure 10c, an added-mass of 0.174 kg was magnetically attached to the top surface of the steel plate to simulate stiffness-loss scenarios. The added-mass was a cylinder-shaped block with a height of 0.02 m, a diameter of 0.04 m, a weight of 0.174 kg. For the added-mass attachment, a 2-cm grid with 60 intersections was designed over a quarter of the splice plate, as plotted in Figure 10c. Totally, 61 scenarios (i.e., an intact and 60 added-mass scenarios) were conducted during the experiment. To avoid uncertain errors caused by the temperature change, the impedance data should be measured in a short period of time under constant temperatures. Therefore, the impedance signal of the PZT interface was repeatedly measured five times for each scenario and the room temperature was controlled as near-constant of 22.3 °C by air-conditioners (see Figure 10d).

### 4.2. Experimental Characterization of Sensing Region on Connection Splice Plate

#### 4.2.1. Experimental Impedance Signatures

**Figure 11:**
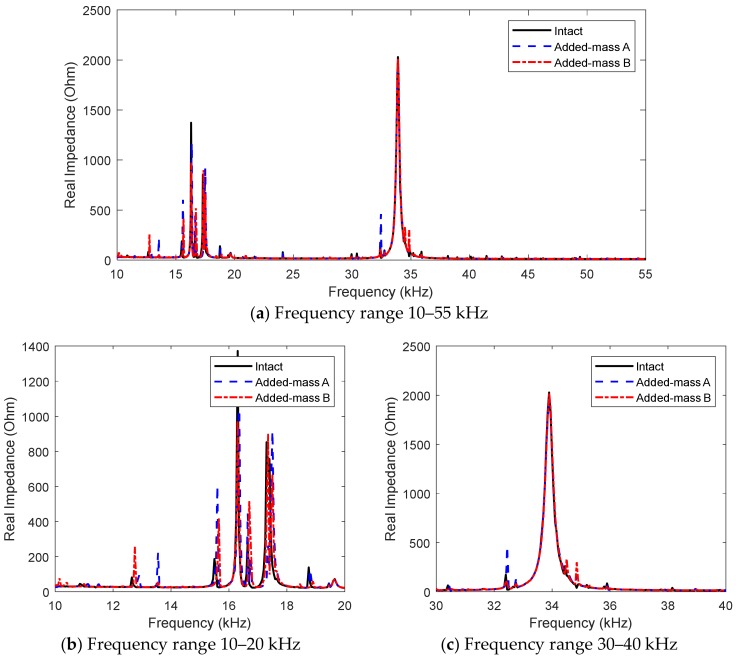
Experimental impedance signatures of PZT interface for intact and damage cases of connection splice plate.

Figure 11 shows experimental impedance signatures in 10–55 kHz of the PZT interface for the intact state and added-masses A and B. Within 10–55 kHz, there exist two major resonant ranges including 10–20 kHz and 30–40 kHz, as respectively plotted in Figure 11b,c. As compared with the FE results in Section 3.2, the experimental impedance signatures contain several additional resonant peaks. This is mainly due to the different sizes of the plate domain. As observed from Figure 11b,c, the resonant impedance responses significantly varied along with the location of the added-mass on the splice plate. The impedance in the first resonant range (10–20 kHz) showed more changes than that in the second one (30–40 kHz).

#### 4.2.2. Sensitivity of Experimental Impedance Features with Respect to Damage Location

**Figure 12:**
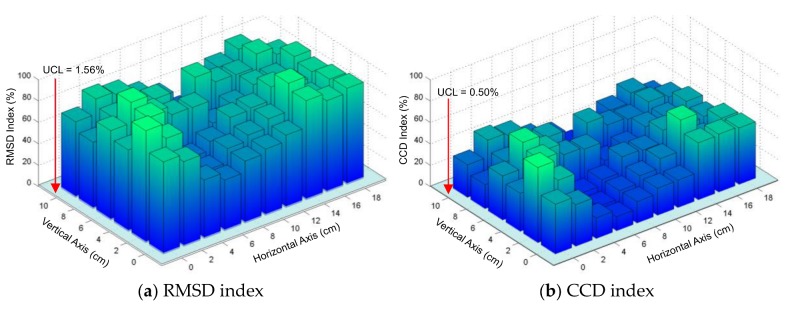
Damage detectability of PZT interface over a quarter of connection splice plate: 10–20 kHz.

**Figure 13:**
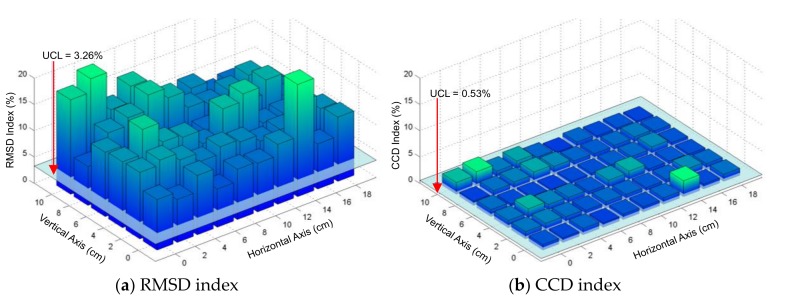
Damage detectability of PZT interface over a quarter of connection splice plate: 30–40 kHz.

The damage-sensitive features (i.e., RMSD and CCD) were extracted from the impedance signatures under all damage cases. Figure 12a,b show the RMSD and CCD indices of the frequency range 10–20 kHz, which were plotted over a quarter of the splice plate. For decision-making on damage occurrence, the UCL thresholds were computed using Equation (5). The uncertain conditions in the experiment could include effects of minor temperature variation (see Figure 10d), and measurement errors of the impedance analyzer. As observed in Figure 12a,b, the RMSD and CCD indices were significantly higher than the UCL thresholds, suggesting that all damages were well detected. Figure 13a,b show the RMSD and CCD indices of the frequency range of 30–40 kHz, which were plotted over a quarter of the splice plate. As observed in the figures, the RMSD index was significantly higher than the UCL threshold; meanwhile, the CCD index was slightly above the UCL threshold. The observations from Figure 13 suggest that all damages were well detected. By comparing the magnitudes of damage-sensitive indices (RMSD and CCD), it is clear that the frequency range of 10–20 kHz was much more sensitive to the damage than that of 30–40 kHz.

From Figure 12 and Figure 13, it is found that the damage locations close to the vertical axis (*θ* ≈ 90°) resulted in relatively higher magnitudes of RMSD and CCD indices than other locations. This experimental result was agreed with the previous numerical observation on the plate domain in Section 2.2. However, the damage locations at near the boundaries of the splice plate were also well detected. The difference can be caused by the limited boundaries of the splice plate. In the next section, the numerical simulation is conducted to confirm the experimental results.

**Figure 14:**
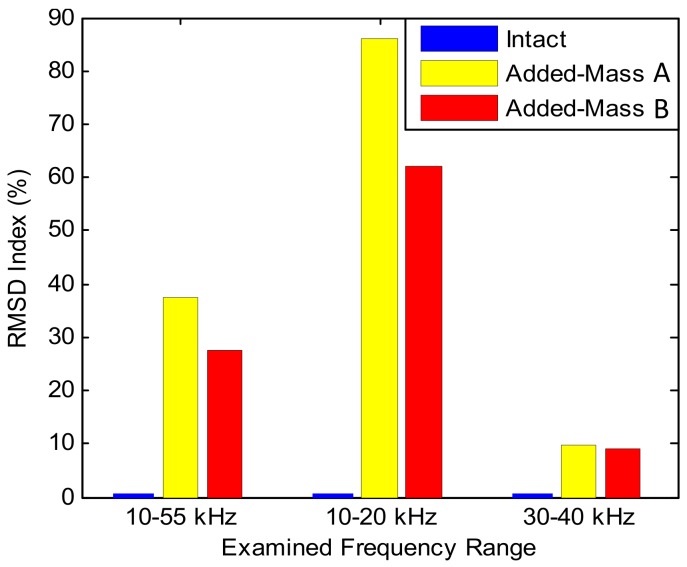
Experimental impedance features (RMSD indices) of PZT interface under damage cases in connection splice plate.

The experimental impedance features (RMSD indices) under the intact and the added-masses A and B were extracted in Figure 14. The location of the added-masses A and B was shown in Figure 10c. It is observed that the RMSD magnitudes was significantly changed when the mass was added to the splice plate. The magnitudes of the RMSD indices under the added-mass A (located at the center of the tested plate) was lower than those under the added-mass B (located at about a quarter of the splice plate). Among three examined frequency ranges (i.e., 10–55 kHz, 10–20 kHz, 30–40 kHz), the frequency range of 10–20 kHz showed the highest sensitivity to the added-mass scenarios.

### 4.3. Numerical Validation of Sensing Region on Connection Splice Plate

#### 4.3.1. FE Modeling

**Figure 15:**
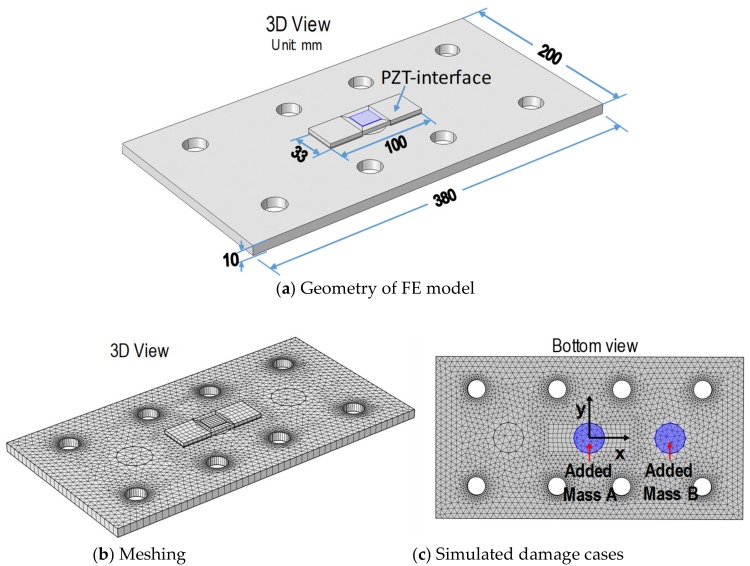
FE modeling for analyzing sensing region of PZT interface on a connection splice plate.

To support the previous experimental results on the sensing region analysis of the PZT interface over the connection splice plate, an FE analysis was conducted. The FE modeling of the PZT interface-splice plate system was established using COMSOL Multiphysics. Figure 15a shows the detailed geometry of the FE model that was in accordance with the previous experiment model in Section 4.1. In FE modeling, the steel splice plate, the aluminum interface, and the PZT patch were discretized by elastic solid elements in 3D, as shown in Figure 15b. The bonding layers of the PZT sensor and PZT interface were simulated and also discretized by the solid elements. A complete mesh of the FE model consists of 5466 solid elements. Since four sponge blocks supporting the experimental model have ignorable elastic stiffness as compared to the steel plate, they were not simulated and free boundary conditions were assigned to the splice plate. The piezoelectric properties of the PZT-5A patch, can be found in Huynh et al. [31]. The material properties of the steel plate, the aluminum interface are listed in Table 1.

The properties of the bonding layers were selected as follows: mass density of 1700 kg/m^3^, Poisson’s ratio of 0.38, Young’s modulus of 6 GPa, and thickness 0.1 mm (see Section 3.1). Figure 15c shows two locations of an added-mass simulated in the FE model. The added-masses A and B in the previous experiment (see Figure 10c) were simulated in the FE model. The added-mass A located at the center while the added-mass B was eccentrically assigned at about a quarter of the splice plate. The added-mass weights 0.174 kg, which was assigned to the circular area (diameter of 0.04 m) on the splice plate (see Figure 15c). A harmonic voltage with an amplitude of 1V was applied to the PZT patch for acquiring the EM impedance. The impedance responses of the PZT interface were numerically analyzed for the intact case and the two simulated added-mass cases. The frequency range of impedance signatures was 10–55 kHz (901 interval points).

#### 4.3.2. Numerical Impedance Signatures

**Figure 16:**
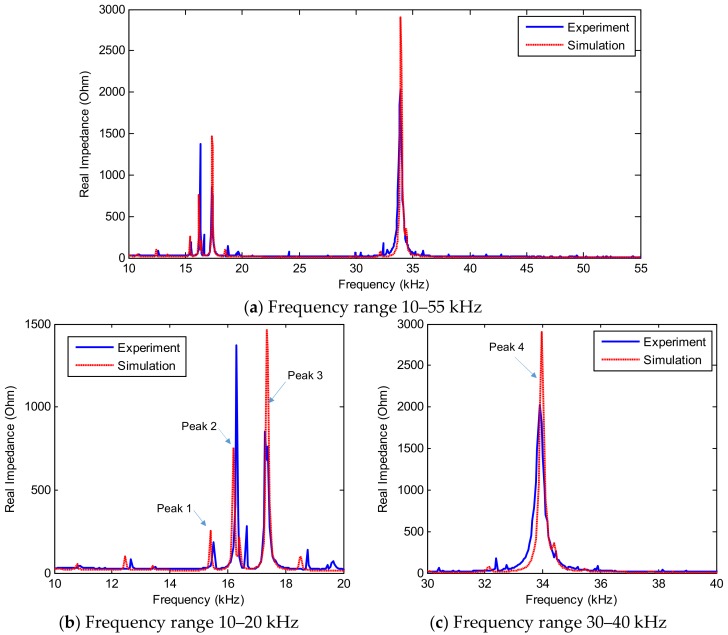
Real impedance signatures of PZT interface under intact case of connection splice plate: simulation versus experiment.

**Figure 17:**
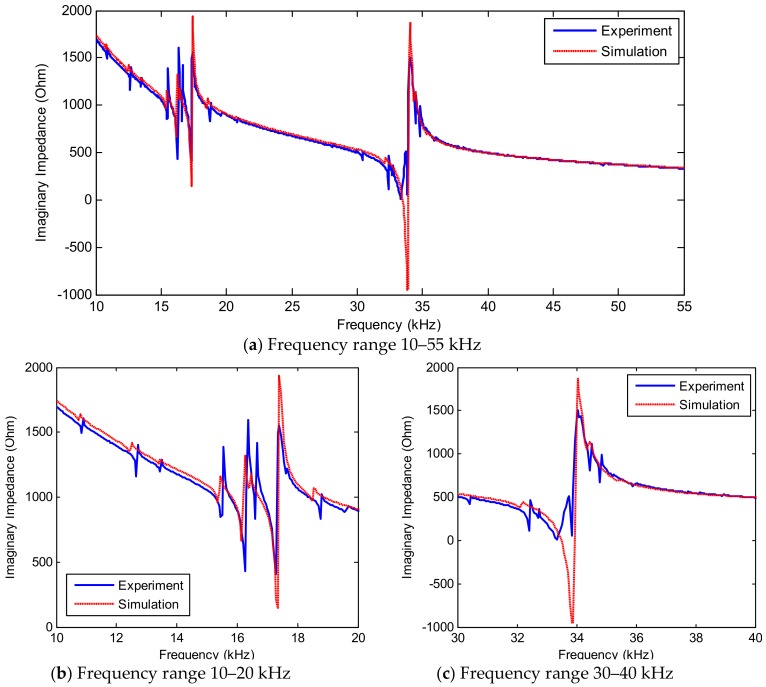
Imaginary impedance signatures of PZT interface under intact case of connection splice plate: simulation versus experiment.

The numerical impedance signatures obtained from the FE model were compared with those from the experimental test. Figure 16 shows the comparison of the real impedance signatures in the frequency range of 10–55 kHz. Two major resonant ranges in 10–20 kHz and 30–40 kHz are zoomed in Figure 16b,c, respectively. Although there were slight differences in the magnitudes of the impedance peaks, the resonant frequencies (i.e., Peaks 1–4) of the numerical simulation were well consistent with those obtained from the experiment at the same frequency ranges and also identical patterns. Figure 17a shows the imaginary part of the numerical impedance response under the intact case. Similar to the real part, the imaginary part of the numerical impedance was also well-matched with the measured impedance signal, see Figure 17b,c.

**Figure 18:**
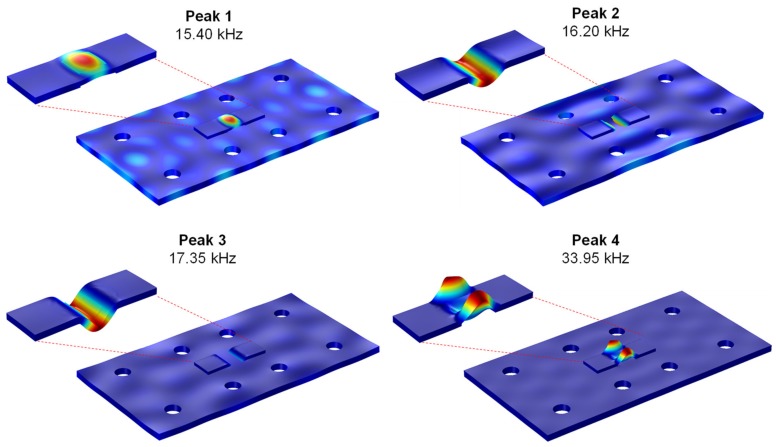
Coupled vibration modes between PZT interface and connection splice plate.

To explain the experimental sensing region of the smart PZT interface on the limited plate domain like the connection splice plate, the coupled dynamics between the PZT interface and the splice plate were computed. Figure 18 shows the mechanical deformation of the splice plate numerically generated by the PZT sensor at four resonant impedance peaks: Peak 1 at 15.40 kHz, Peak 2 at 16.20 kHz, Peak 3 at 17.34 kHz, and Peak 4 at 33.95 kHz. The piezoelectric deformations of the interface corresponding to Peaks 1–3 were the longitudinal flexural motion while that for Peak 4 was the lateral flexural motion. Under the flexural motions of Peaks 1–4, it is found that whole area of the splice plate was excited by the high-frequency waves. Hence, a structural change occurred in any locations on the splice plate would be sensitively detected by the variation of impedance signatures. The coupled vibrations occurred strongly for Peaks 1–3 (in 10–20 kHz) but less for Peak 4 (in 30–40 kHz). This indicates that the first resonant range 10–20 kHz was more sensitive to the change in dynamic properties of the splice plate than that of 30–40 kHz.

#### 4.3.3. Sensitivity of Numerical Impedance Features with Respect to Damage Location

**Figure 19:**
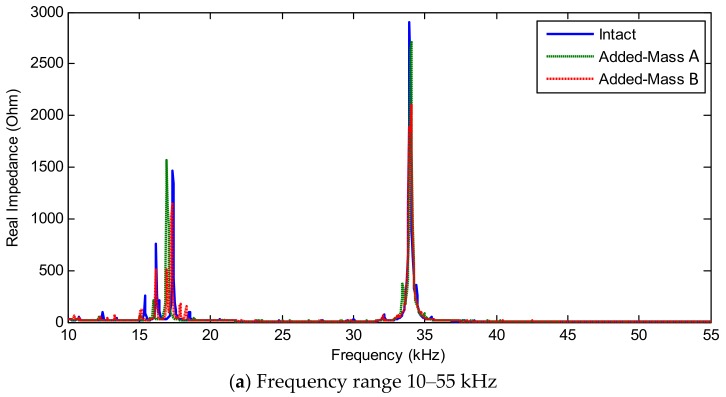

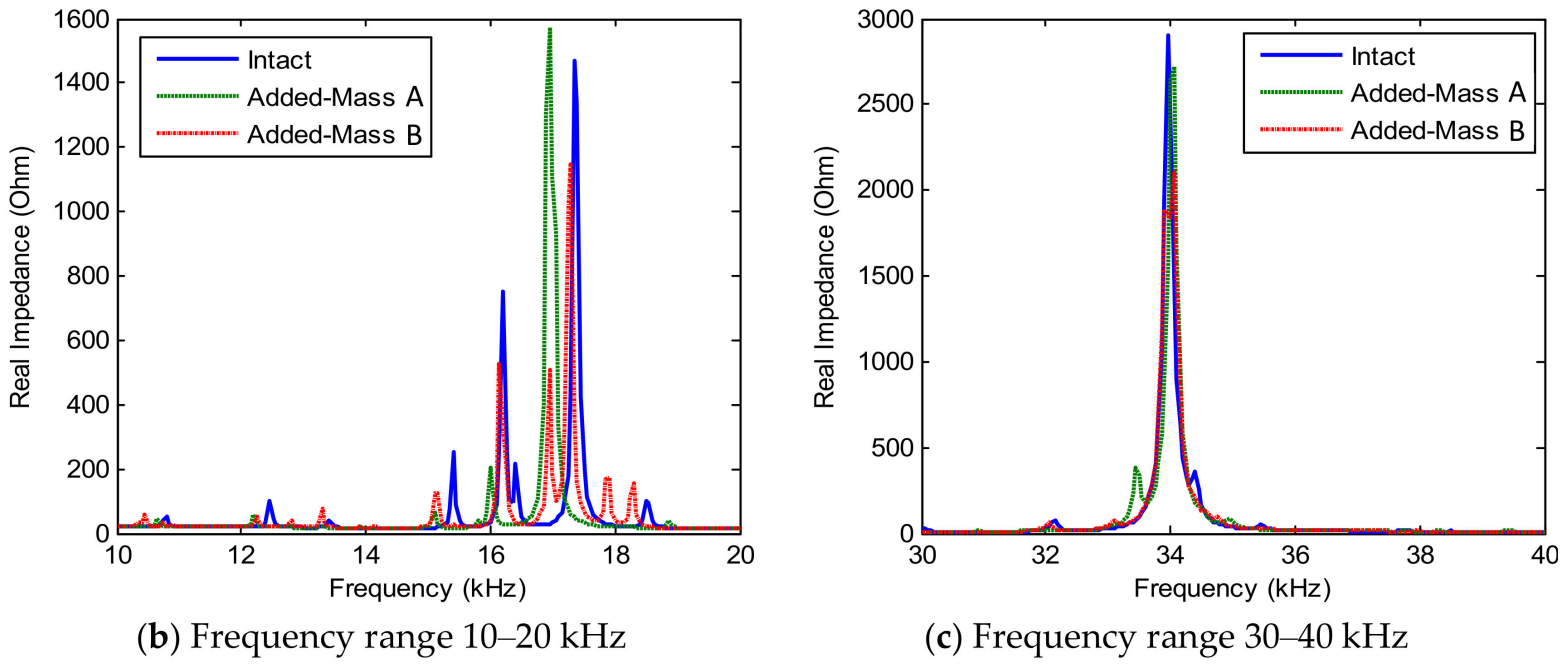
Variations in numerical impedance signatures of PZT interface under damage cases in connection splice plate.

Figure 19a–c respectively show the variations in the real numerical impedance under the damage cases of the splice plate for the frequency ranges of 10–55 kHz, 10–20 kHz, and 30–40 kHz. It is observed that Peaks 1–3 in 10–20 kHz tended to sensitively vary when the mass was added to the splice plate. The frequency shift of Peak 4 in 30–40 kHz was relatively small due to the low level of coupled vibration between the interface and the splice plate, as observed in Figure 18.

**Figure 20:**
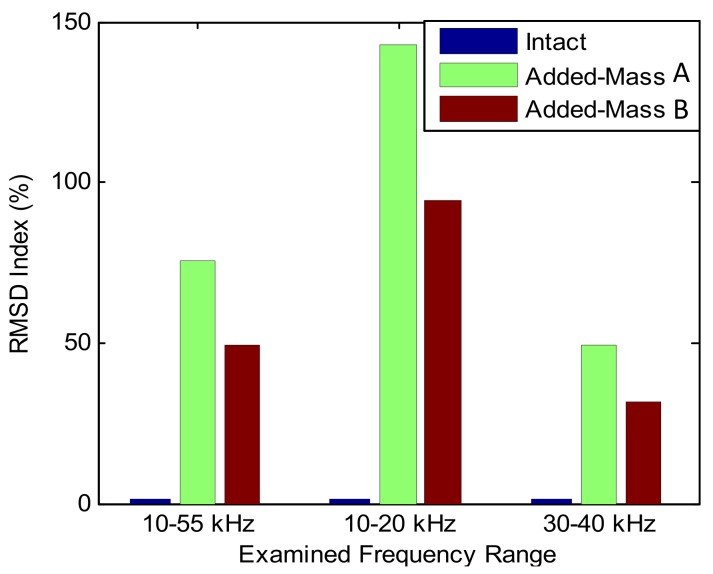
Numerical impedance features (RMSD indices) of PZT interface under damage cases in connection splice plate.

The RMSD indices of the numerical impedance signatures were computed for three examined frequency ranges (i.e., 10–55 kHz, 10–20 kHz and 30–40 kHz). As shown in Figure 20, the RMSD indices were significantly changed when the added-masses A and B were assigned on the splice plate. Among three examined frequency ranges, the frequency range of 10–20 kHz had the most significant magnitudes of the damage indices. The magnitudes of the damage indices under the added-mass A (located at the center of the tested plate) was lower than those under the added-mass B (located at about a quarter of the splice plate). The numerical detection results of the added-masses A and B in Figure 20 showed consistency with the experimental results in Figure 14.

## 5. Conclusions

This study presented numerical and experimental analyses for characterizing the sensing region of a smart PZT (lead–zirconate–titanate) interface for damage monitoring in plate-like structures. First, a finite element (FE) model of the PZT interface mounted on a plate structure was established. The impedance responses of the PZT interface were numerically simulated under different damage locations inflicted in the plate domain. The impedance features were extracted from the impedance signatures to analyze the sensing distance and the damage detectability of the PZT interface. Next, the splice plate of a bolted connection was selected as a practical structure for the experimental evaluation of the PZT interface’s sensing region on a limited plate domain. The damage sensitivity behavior of the PZT interface was analyzed with respect to the damage location on the splice plate. An FE analysis of the corresponding PZT interface-splice plate system was also conducted to support the experimental results.

From the experimental and numerical observations, at least five concluding remarks can be drawn for the sensing region characteristics of the PZT interface, as follows:

(1) The sensing region characteristics of the PZT interface on the large plate domain was different with the limited plate domain such as the rectangular splice plate.
(2) For the circular plate domain, the detectable distance of the PZT interface analyzed by RMSD was about five times of the interface’s length. The detectable orientation *θ* was within (45° to 135°) and (225° to 315°) for the range 10–20 kHz; and (75° to 105°) and (255° to 295°) for the range 30–40 kHz.
(3) For the limited plate domain like the connection splice plate, the detectable zones of the PZT interface were the entire plate. The damage locations near the boundaries of the splice plate or at the angle *θ* ≈ 90° resulted in significant magnitudes of impedance features.
(4) The numerical analyses of the coupled vibration modes of the PZT interface-host structure system can reveal an overall view on the detectable zones over the plate domain.
(5) The frequency range containing the longitudinal flexural motion of the interface device (i.e., 10–20 kHz) had significant sensing regions.

As the future study, the effects of environmental factors and damage-type on the sensing region characteristics of the PZT interface should be analyzed extensively. There is also a need to fine-tune the design of the interface to minimize the unexcited regions on the plate domain.

## Figures and Tables

**Table 1:** Mechanical properties of interface, circular plate and bonding layer [31].

Parameters	Aluminum Interface	Steel Circular Plate	Bonding Layer
Young’s modulus, *E* (GPa)	70	200	6
Poisson’s ratio, *υ*	0.33	0.33	0.38
Mass density, *ρ* (kg/m^3^)	2700	7850	1700
Damping loss factor, *η*	0.004	0.004	0.004
